# Commentary: Building the Older Adult Fall Prevention Movement – Steps and Lessons Learned

**DOI:** 10.3389/fpubh.2016.00277

**Published:** 2016-12-22

**Authors:** Matthew Lee Smith, Sofia Chaudhary, Sharon Nieb, Rana Bayakly, Kathleen Graham, Elizabeth Head

**Affiliations:** ^1^Department of Health Promotion and Behavior, College of Public Health, Institute of Gerontology, The University of Georgia, Athens, GA, USA; ^2^Department of Health Promotion and Community Health Sciences, Texas A&M School of Public Health, College Station, TX, USA; ^3^Emory University School of Medicine, Division of Pediatric Emergency Medicine, Children’s Healthcare of Atlanta, Atlanta, GA, USA; ^4^Injury Prevention Research Center at Emory, Department of Emergency Medicine, Emory University School of Medicine, Atlanta, GA, USA; ^5^Georgia Department of Public Health, Atlanta, GA, USA; ^6^School of Occupational Therapy, Brenau University, Gainesville, GA, USA

**Keywords:** fall prevention, fall prevention movement, coalitions, older adults, partnerships

Coalitions are powerful systems change agents because of their ability to unite sets of diverse organizations and multidisciplinary professionals around a particular issue to support action and policy (1). In this context, Schneider and Beattie (2) discussed the importance of building, and steps taken to establish, a national movement to prevent falls and fall-related injuries among older adults in the United States. As part of this movement to combat fall incidence rates and the ramifications of injurious falls, the FallsFree^®^ Initiative (3) supports 42 State Fall Prevention Coalitions (SFPC) to address falls and related risk factors by the following: (A) identifying and promoting the issue; (B) engaging partners and leaders; and (C) identifying solutions (2). Alongside, this established and growing national effort to organize fall prevention advocacy, action, and policy at the state level (2) and localized coordinated activities within states serve as a niche to incite cross-disciplinary collaboration to improve older adult health through innovative solutions.^1^ This commentary focuses on a within-state task force with the potential to complement efforts of the Georgia SFPC to prevent falls.

Founded in 1993, the Injury Prevention Research Center at Emory (IPRCE) is a collaborative, multi-institution research center housed within Emory University’s School of Medicine, Department of Emergency Medicine. The goal of IPRCE is to reduce the burden of violence and unintentional injuries, which is accomplished through the missions of five distinct task forces comprised of diverse professionals and disciplines representing universities, public agencies, private organizations, and community stakeholders. The five task forces include the following: (A) Fall Prevention, (B) Drug Safety, (C) Traumatic Brain Injury (TBI)/Concussion Prevention, (D) Transportation Safety, and (E) Violence Prevention. Each task force works regularly and closely with a common Associate Director of Programs. IPRCE supports all task force initiatives by assisting with organization, establishing connections, assisting to identify funding sources, and providing expertise with respect to research, training, and education, community outreach, and policy.

Nationally, it is well recognized that falls among older adults are a growing public health concern because of their prevalence and ramifications associated with injury, morbidity, loss of independence, premature mortality, and societal costs (4–7). These same issues persist in the state of Georgia. Figure 1 presents statewide fall-related emergency room (ER) visit data by age and sex. As can be seen, fall-related ER visits are substantially higher among adults aged 70 years and older, with females experiencing a disproportionate burden. However, age-based fall-related ER visits are multimodal, with youth between the ages of 1 and 4 years experiencing disproportionately high ER visit rates (actually rivaling ER visit rates of those aged 70–79 years).

**Figure 1:**
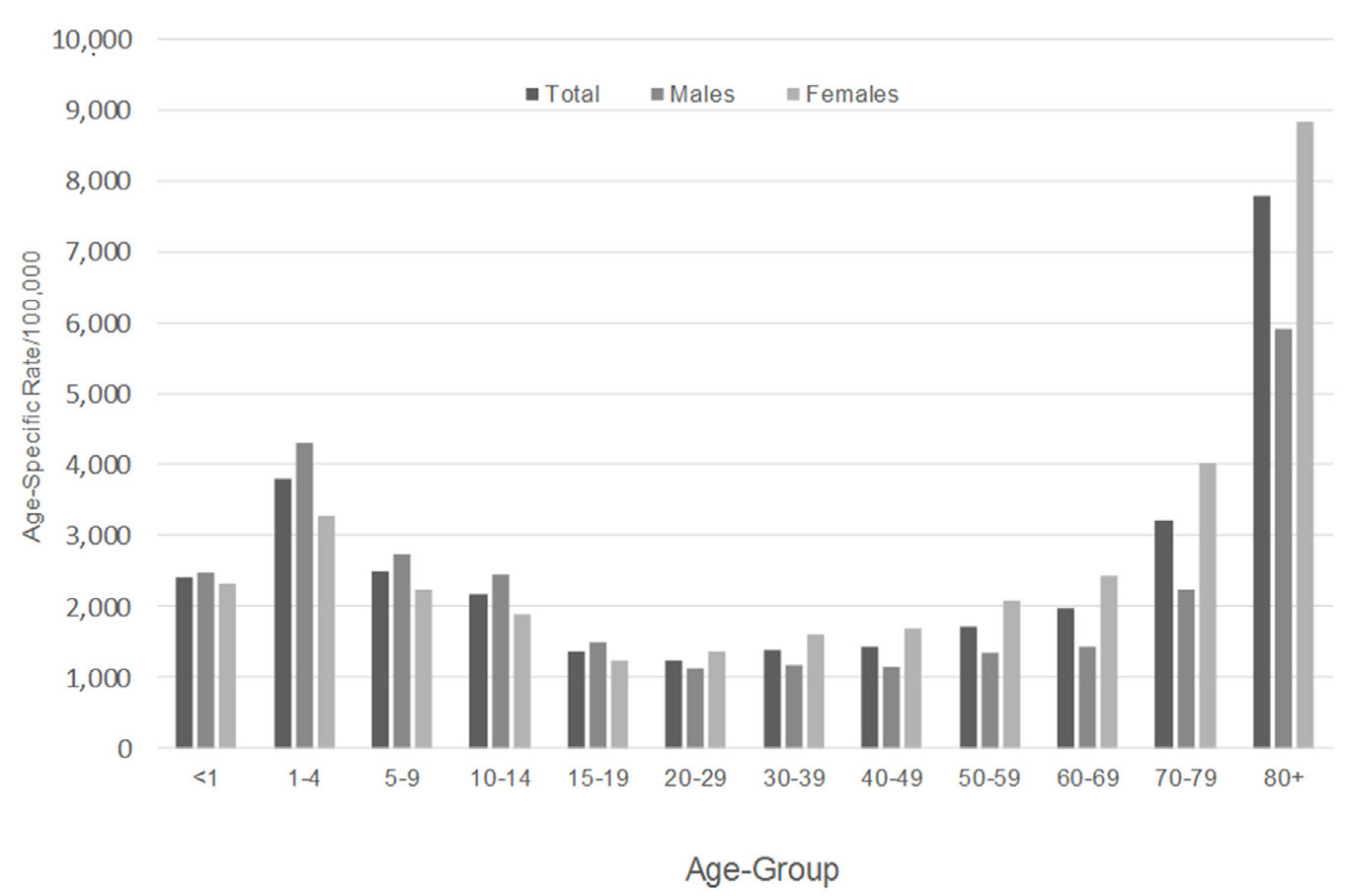
**Falls-related emergency room visits by sex and age groups, Georgia 2014**.

Given this “u-shaped” trend for fall-related ER visits in Georgia, the IPRCE Fall Prevention Task Force addresses falls across the lifespan continuum, with special emphases on older adults and infant/toddlers. The goals of the task force are to (A) set the goals and priorities for the task force based on the regional, state, and local data; (B) identify research gaps and guide studies that will address root causes of falls within the community; (C) develop a strategic plan to address research/service gaps, which involve the community as collaborators; (D) assist with the implementation of recommended interventions; (E) set specific deadlines and metrics for documenting success; and (F) provide consultation about the development and evaluation of evidence-based fall prevention programs.

Following these task force goals and driven largely by the data presented in Figure 1, the IPRCE Fall Prevention Task Force comprised approximately 15 dedicated professionals representing universities, hospitals and health-care systems, non-profit organizations, the Georgia Department of Public Health (i.e., lead of the Georgia Fall Prevention Coalition), and the Centers for Disease Control and Prevention. More broadly, IPRCE’s emphasis on injury prevention creates opportunities for natural collaboration between its five task forces. These collaborations organically develop around falls because falls are strongly associated with other task force issues (most notably, drugs/medication, TBI/trauma, violence, and transportation).

The dual population foci of this task force foster innovation to address falls among older adults. For example, intergenerational approaches to address falls are emerging, which primarily target the “sandwich generation” (8) as change agents for their older adult parents. However, opportunities exist to develop interventions that target the “club sandwich generation” as triple change agents for fall prevention among their own infants/toddlers, middle-aged parents, and older adult grandparents. This Fall Prevention Task Force is exploring such intervention options for development and delivery internal and external to the clinical setting.

Given the array of multilevel evidence-based programs and solutions to address older adult falls, the need for this Fall Prevention Task Force to develop new interventions is diminished. Instead, this task force recognizes that multi-level fall prevention efforts often occur in silos (9), and integrating interventions across community and clinical settings remains complex (10). It focuses on improving and enhancing fall prevention intervention/service connectivity across community sectors. Based on the specified training of the task force members, and the organizations they represent, this task force places emphasis on supporting innovative models to integrate elements of the STopping Elderly Accidents, Deaths, and Injuries (STEADI) tool kit into community screenings, emergency medical service first responder training/practice, and programs such as the Otago Exercise Program and A Matter of Balance. Furthermore, this task force will strive to identify ways to facilitate “real time” communication and promote seamless referrals between silos and sectors.

To conclude, the IPRCE’s mission to address injury in Georgia explicitly targets fall prevention for children and older adults as a complement to existing Georgia Fall Prevention Coalition efforts. This task force is emerging as the advisory group for science and innovation for fall prevention in the state. Through this collaborative, efforts to serve older Georgians with fall prevention interventions and resources will be complemented with research and evaluation expertise. This model to complement SFPC has vast implications for replicability in other states and has potential for strategic planning and leveraging efforts to expand funding for fall prevention statewide.

## Author Contributions

MS, SC, SN, RB, KG, and EH were involved in the writing and reviewing of the manuscript.

## Conflict of Interest Statement

The authors declare that the research was conducted in the absence of any commercial or financial relationships that could be construed as a potential conflict of interest.

## Footnotes

^1^Smith ML, Schneider EC, Byers IN, Shubert TE, Wilson AD, Towne SD Jr, et al. Systems change and sustainability associated with multi-faceted evidence-based fall prevention efforts in three states. (unpublished, *Front Public Health*, 2016).
